# Early-life stress impairs acquisition and retrieval of fear memories: sex-effects, corticosterone modulation, and partial prevention by targeting glucocorticoid receptors at adolescent age

**DOI:** 10.1016/j.ynstr.2024.100636

**Published:** 2024-04-23

**Authors:** Jeniffer Sanguino-Gómez, Harm J. Krugers

**Affiliations:** Brain Plasticity Group, SILS-CNS, University of Amsterdam, Amsterdam, the Netherlands

**Keywords:** Early-life stress, Fear, Memory, Corticosterone, Glucocorticoid antagonist

## Abstract

The early postnatal period is a sensitive time window that is characterized by several neurodevelopmental processes that define neuronal architecture and function later in life. Here, we examined in young adult mice, using an auditory fear conditioning paradigm, whether stress during the early postnatal period 1) impacts fear acquisition and memory consolidation in male and female mice; 2) alters the fear responsiveness to corticosterone and 3) whether effects of early-life stress (ELS) can be prevented by treating mice with a glucocorticoid (GR) antagonist at adolescence. Male and female mice were exposed to a limited nesting and bedding model of ELS from postnatal day (PND) 2–9 and injected i.p with RU38486 (RU486) at adolescent age (PND 28–30). At two months of age, mice were trained in the fear conditioning (FC) paradigm (with and without post training administration of corticosterone - CORT) and freezing behavior during fear acquisition and contextual and auditory memory retrieval was scored. We observed that ELS impaired fear acquisition specifically in male mice and reduced both contextual and auditory memory retrieval in male and female mice. Acute post-training administration of CORT increased freezing levels during auditory memory retrieval in female mice but reduced freezing levels during the tone presentation in particular in control males. Treatment with RU486 prevented ELS-effects in acquisition in male mice and in females during auditory memory retrieval. In conclusion, this study highlights the long-lasting consequences of early-life stress on fear memory processing and further illustrates 1) the potential of a glucocorticoid antagonist intervention during adolescence to mitigate these effects and 2) the partial modulation of the auditory retrieval upon post training administration of CORT, with all these effects being sex-dependent.

## 1 Introduction

The early postnatal period is a sensitive time window that is characterized by several neurodevelopmental processes that shape brain development and brain function (Chen and Baram, 2016; Fox et al., 2010; Luby et al., 2020; McEwen, 2008; Webb et al., 2001). Such changes include synaptogenesis, synaptic pruning, neurogenesis and dendritic and axonal arborization that determine neuronal and network function and behavior later in life (Kolb et al., 2013, 2017). These dynamic changes also render the brain vulnerable to adverse environmental factors, such as early life adversity. In humans, early life adversity includes maltreatment, emotional neglect, abuse and undernutrition, that can have persistent effects on child development (Smith and Pollak, 2020). In rodents (mice and rats), models of early life adversity often involve stressful disturbances that arise from disturbed levels of maternal care during the early postnatal period. Studies on ELS in rodents indicate that limited or fragmented levels of maternal care during the early postnatal period can persistently impair hippocampal synaptic function (for a systematic review see Bonapersona et al., 2019; Rocha et al., 2021), spatial (Bolton et al., 2020; Kanatsou et al., 2017; Molet et al., 2016; Naninck et al., 2015) and contextual memory (Arp et al., 2016; Lesuis et al., 2019; Manzano Nieves et al., 2020) and alter emotional memory regulation (Walker et al., 2017). These effects of early life adversity on behavior and memory are often sex dependent (Loi et al., 2014, 2017a; Oomen et al., 2009; Parel and Peña, 2022; Perry et al., 2021; van Hasselt et al., 2012) with studies in rodents suggesting that females are generally more resilient than males towards behavioral effects of ELS (Arp et al., 2016; Bath et al., 2017; Naninck et al., 2015).

One possible explanation for ELS effects later in life is that it increases the sensitivity to stressors (Balouek et al., 2023; Bolton et al., 2019; Liu et al., 1997; van Bodegom et al., 2017). This may be mediated by glucocorticoid hormones (GCs - cortisol in humans, corticosterone in rodents) that are released in elevated levels from the adrenal glands after stress-exposure (Ceruso et al., 2020; Orso et al., 2020; van Bodegom et al., 2017; Walker et al., 2017). Consistent with this idea, modulation of the stress signalling can prevent (sometimes partly) ELS effects on memory later in life, for example by genetic ablation and blocking corticotropin releasing hormone (CRH) receptors or pharmacologically blocking mineralocorticoid receptors and glucocorticoid receptors (MRs and GRs respectively) (Arp et al., 2016; Bolton et al., 2022; Ivy et al., 2010; Kanatsou et al., 2017; Loi et al., 2017b; Short et al., 2020). Some of these interventions were implemented during adolescence (Arp et al., 2016; Loi et al., 2017b), which may serve as a “window of opportunity” due to the brain's high degree of plasticity during this developmental stage (Dorn et al., 2019; Méndez Leal and Silvers, 2021).

In the brain, GCs, MRs and GRs regulate neuronal function via non-genomic and genomic pathways (de Kloet and Joëls, 2023). Via these actions, GCs regulate synaptic function (Popoli et al., 2012; Sanacora et al., 2022) and memory processes including memory consolidation, memory retrieval and memory generalization (Bahtiyar et al., 2020; Brosens et al., 2023a, Brosens et al., 2023b; dos Santos Corrêa et al., 2021; Hui, 2004; Kaouane et al., 2012; Lesuis et al., 2021; McReynolds et al., 2010; Quirarte et al., 2009). While cellular studies suggest that ELS increases the sensitivity of synapses to GCs (Champagne et al., 2008; Pillai et al., 2018) relatively little is known whether/how ELS alters the sensitivity to glucocorticoids at the behavioral level.

Therefore, the present study was set out to test whether and how ELS, induced by housing dams and offspring with limited nesting and bedding material between PND 2–9 alters fear acquisition and memory (in both male and female mice) and its modulation by post training administration of corticosterone. Finally, we tested whether pharmacological targeting of GRs using the antagonist RU486 at adolescence was effective in altering ELS effects on memory later in life.

## 2 Methods and materials

### 2.1 Animals

**Fig. 1:**
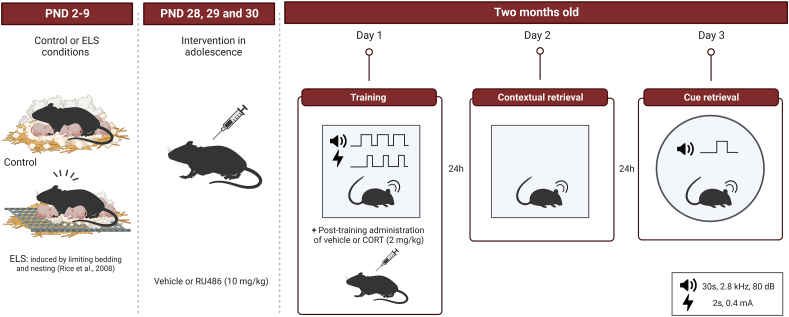
Experimental design and timeline of behavioral experiments. Schematic overview of the auditory fear conditioning paradigm. Male and female mice were exposed to either control or ELS from PND 2–9. During adolescence (PND 28–30), mice received injections with vehicle or the glucocorticoid receptor (GR) antagonist RU486 (10 mg/kg). At PND 60, the mice underwent training in a mild auditory fear conditioning task, followed by an intraperitoneal injection of CORT (2 mg/kg) or vehicle after training in context A. 24 h later, contextual memory was evaluated and 24 h after that, auditory was tested in context B. Created with BioRender.com.

Mice were bred and housed under standard conditions at a room temperature of 20–22 °C and a humidity of 40–60%. Mice were kept on a 12/12 h light/dark (lights on from 8:00 a.m. to 8:00 p.m.) and a radio providing background noise continuously. The animals were fed *ad libitum* with standard lab chow and water. All experiments were conducted during the light phase and were approved by the animal Welfare committee of the University of Amsterdam and conducted under Dutch national law and in compliance with the EU directive 2010/63/EU. An overview of the experimental design is shown in Fig. 1.

### 2.2 Breeding

As this study was part of a larger overarching project, animal cohorts were established with a transgenic mouse model that has a fluorescent reporter gene under the promotor of the immediate early gene Arc (ArcdVenus mice, kindly provided by prof. dr. Steven Kushner, Erasmus University Rotterdam) (Brosens et al., 2023a; Eguchi and Yamaguchi, 2009; Lesuis et al., 2021, Rao-Ruiz et al., 2019).

In order to standardize the perinatal environment, mice were bred in-house. Six to eight-week-old female C57BL/6J mice were purchased from Envigo Laboratories (Venray, Netherlands), and allowed to habituate for two weeks before breeding. After acclimatization, two females and one ArcdVenus homozygote male mouse (Brosens et al., 2023a, Brosens et al., 2023b; Eguchi and Yamaguchi, 2009; Lesuis et al., 2021) were housed together in a conventional type II cage with standard bedding and nesting and paper enrichment for one week to ten days to allow mating. We used wild-type females for breeding to avoid potential line-dependent changes in maternal care. Next, the female mice were housed together for another week with standard bedding and small amount of the old one to keep the odour, half the amount of paper cage enrichment and a 5 × 5 cm square of cotton nesting material (Tecnilab-BMI, Someren, The Netherlands). After another week of paired-housing, pregnant primiparous females were housed individually in a conventional type II cage covered with a filtertop, normal bedding, one nestlet and a small amount of old bedding material, with no paper cage enrichment. The animals were moved to a different room starting from 18 days after the start of the breeding and monitored daily for the birth of the pups. Dams were checked for the birth of pups each morning before 09:00 a.m. When a litter was born, the previous day was designated as PND 0.

### 2.3 Early life stress paradigm

The early life stress paradigm consisted of housing mice with limiting nesting and bedding (LBN) material between PND 2 to PND9 as described previously (Lesuis et al., 2019; Naninck et al., 2015; Rice et al., 2008). At PND 2, litters were culled to 6 pups per litter. Litters of less than 5 pups or litters with only one sex were excluded. Dams and their litters were randomly assigned to the ELS or control condition until PND 9, after which all mice were treated equally. The control group was housed with a standard amount of sawdust (≈100g) and a standard 5 × 5 cm square of cotton material (Tecnilab-BMI, Someren, The Netherlands). For the ELS paradigm, the dams were provided with a reduced amount of sawdust (≈33g, 1/3 of the standard amount) and half of the standard square of cotton material (2.5 × 5 cm). In addition, a stainless-steel grid (22 cm × 16.5 cm, mesh size 5 mm × 5 mm, in-house made) was placed 1 cm above the cage floor to prevent the use of sawdust for nesting. Filtertops remained for both conditions. Both groups were not disturbed until PND 9 when they were weighted and put back into standard housing, in which they remained till PND 21, when they were weaned. At that age, ear clips were taken for identification and to determine their genotype.

### 2.4 RU486 and corticosterone treatment

Mice from both the control and ELS group received intraperitoneal vehicle (0.25% caboxymethylcellulose, 0.1% Tween in saline) or RU486 (Sigma-Aldrich M8046, dissolved in vehicle; final dose 10 mg/kg, injection volume 5 μl/g body weight - Arp et al., 2016) injections. RU486 and vehicle were given daily on PND 28, PND 29 and PND 30 before 9:00 a.m. to avoid circadian elevations in corticosterone levels.

Corticosterone (CORT, Sigma-Aldrich C2505 (16 mg/ml dissolved in 99.9% EtOH and diluted 40x in saline; final dose: 2 mg/kg, injection volume 5 μl/g body weight - Brosens et al., 2023a; Lesuis et al., 2021) or the corresponding vehicle solution were injected intraperitoneally immediately following training in the fear conditioning paradigm.

### 2.5 Fear conditioning

Two months (±2 weeks) old male and female mice were tested in an auditory fear conditioning paradigm. One week prior to fear conditioning, the mice were single housed in isolated cabinets to avoid bias after subsequent testing order, and possible transmission of fear. All behavioral procedures occurred in the morning between 08.00 a.m. and 10.00 a.m. when basal plasma corticosterone levels are low. The fear conditioning protocol was run with a computer with Ethovision software (version 14.0, Noldus, The Netherlands) and mouse behavior was recorded with a camera (*Basler acA1300-30gm* GigE, Ahrensburg, Germany) connected to the computer. On the training day, mice were placed in the conditioning chamber (Context A: 17 cm × 17 cm x 25 cm, Ugo Vasile, with steel grid floor connected to a shock generator) for 180 s with white noise (1.2 kHz, 50 dB) to freely explore the context. Exploration was followed by a Conditioned Stimulus (CS) (30 s, 2.8 kHz, 82 dB) paired to a foot shock (Unconditioned Stimulus, US) during the two last seconds of the tone (2s, 0.4 mA). The tone-shock pairing was presented three times separated by an intertrial interval of 60 s. Sixty seconds after the last footshock, mice were injected with vehicle or CORT and returned to their home cage. Between animals, the chamber was cleaned with 96% ethanol. Twenty-four hours later, mice were placed back into context A with white noise (50 dB) playing in the background for 180 s. One day later, animals were placed in a completely novel context (Context B, 29.5 cm diameter circular context with white background and black stripes) with white noise (50 dB) in which the animals could freely explore for 180 s. Next, the CS was presented, followed by 60 s of recovery. Context B was cleaned with 1% acetic acid. Video recordings for behavioral analysis were recorded for the three tasks.

### 2.6 Behavioral analysis

Throughout the experiments, freezing behavior, defined as ‘the absence of movement except the ones required for breathing’ (Zhou et al., 2009), was scored using an automatic procedure, consisting in a deep-learning pose estimation algorithm, Deeplabcut (Mathis et al., 2018), in combination with a machine-learning tool for supervised behavior classification, SimBA (Nilsson et al., 2020). Behavior was scored during training: exploration (first 180 s) the three paired tone-shock (30 s) and the intertrial periods (60 s). Freezing during context retrieval was scored 24 h after training for 180 s. Freezing during auditory cue retrieval was scored 180s before the tone (pre-cue), during the tone-exposure (cue) and 60s after the tone (post-cue). Freezing levels were calculated as percentage of the total time. Locomotor activity was calculated based on the cumulative distance moved by the centroid point of the animal, tracked using Deeplabcut (Mathis et al., 2018) and transformed from pixels to mm.

### 2.7 Estrous cycle analysis

In order to determine the estrous cycle phase, vaginal swabs were taken after completion of the fear conditioning testing in context B and stained using a Giemsa staining in distilled water (1:15) (Cora et al., 2015; Ruigrok et al., 2021).The slides were incubated with the staining solution for 10 min at room temperature. The slides were then rinsed with distilled water 4 times. Finally, the slides were air dried for 5 min. The oestrous phase was determined by using a Zeiss Axioscan brightfield microscope under 4x magnification. The analysis focused solely on comparing the estrous phase to the non-estrus (including proestrus, metestrus and diestrus) phase.

### 2.8 Plasma corticosterone measurements

Blood samples were collected in ice cold, EDTA-coated tubes (Sarstedt, Etten-Leur, The Netherlands), placed on ice and centrifuged at 14.000 rpm for 25 min. This process isolated plasma, which was stored at −20 °C until further analysis. Plasma corticosterone levels were measured using a commercially available test kit (Immunodiagnosticsystems, AC-15F1, Tyne and Wear, United Kingdom).

### 2.9 Statistical analysis

Animals were randomly assigned to condition and treatment, and data analysis was conducted by an experimenter who was blinded to the experimental condition. Sample size was determined a priori by conducting a power analysis with GPower 3.1. Statistical analysis and data visualization was performed using R version 4.2.1 (2022-06-23 ucrt) and RStudio version (2022.7.2.576). Packages used and corresponding citations are summarized in Supplementary Table 1.

Outliers were screened using the interquartile range (IQR) method: data above Q3 + 3xIQR or below Q1 - 3xIQR that had methodological or biological deviations were considered as extreme outliers and therefore, removed from the data. If removed in one of the variables, the outlier was removed from the entire analysis. Normality of the data was assessed using Shapiro-Wilk test, and homogeneity of the variance by Levene's test. Sphericity was assessed using Mauchly test and whenever data doesn't meet these criteria, Greenhouse–Geisser corrected results were used.

Due to the nature of the experiment, the ELS variable is intrinsically bound to a specific litter. Therefore, prior to any analysis, we tested for nested effects by running an ANOVA comparing the use of litter as a random factor. Whenever the test was significant, data was analysed including litter as a random factor in a linear mixed model (Aarts et al., 2014). Similarly, a nested test was done to analyse the contribution of the estrous phase to our results, comparing a model with and without considering estrous cycle as a random factor.

Analysis was performed both combined and separately for male mice and female mice (Supplementary Material and Methods) but the data shown is separated per sex. Single-factor parameters were analysed with *t*-test, either paired (comparison freezing during training versus context) or unpaired (body weights and gain) given the nature of the data. For the locomotor activity and generalization analysis a two-way ANOVA using stress and RU486 treatment as between factors was performed. For the remaining analysis, a 3 × 2 x repeated measures factorial analysis of variance was performed to assess the interaction between three factors (ELS and RU486 and CORT treatments) over time (repeated measures). For that, a mixed model ANOVA was performed with three in between factors (ELS, RU486 and CORT treatments) and one within (time). Mixed model was used although the full data set doesn't meet the assumption of normality since the mixed model is quite robust to violations of this assumption. For interaction effects, post-hoc analysis was performed by subsequent one or two-way ANOVA stratified by interacting variable. P value (≤0.05) was considered statistically significant for all tests, and it was adjusted per number of tests using Bonferroni correction when needed it.

## 3 Results

### 3.1 ELS transiently decreases body gain between PND 2–9, which normalizes at adolescence age

Exposure to LBN condition between PND 2–9 reduced body gain both in male mice (t(27.63) = 7.134, p < 0.001) and female mice (t(34.22) = 9.391, p < 0.001) (Table 1). Body weight gain normalized after returning mice to their standard housing conditions at PND 9 (body gain from PND 9 till adolescence (PND 28–30): males (t(29.67) = −1.405, p = 0.170); females (t(34.03) = −0.612, p = 0.544)). At the age of testing (PND 60), no ELS effects on body weight were present in male mice and female mice. For absolute body weights at PND 2, PND 9 and adolescent age see Supplementary Table 2.

**Table 1:** Body weight was decreased by ELS in a time dependent matter. Table summarizing body weight gain in control and ELS mice during developmental and final body weight (mean ± SEM) prior testing (PND 60). ELS decreased body gain during the stress period -PND 2 to PND 9- that is normalized once returning to control conditions from PND 9 till adolescence. This is also shown at PND 60. Averages per sex and litter are shown in the pre-weaning values due to the impossibility to trace individual pups at this age. Males: N_Control_ = 16, N_ELS_ = 21. Females: N_Control_ = 17, N_ELS_ = 17. *: ELS effect. Effect p < 0.05.

Period	Body weight gain (g) PND2-9				Body weight gain (g) PND 9-Adolescence				Body weight (g) PND60			
Stress	Control		ELS		Control		ELS		Control		ELS	
Sex	Male	Female	Male	Female	Male	Female	Male	Female	Male	Female	Male	Female
	3.05 ± 0.10	3.19 ± 0.13	1.82 ± 0.08 (*)	1.95 ± 0.11 (*)	10.19 ± 0.37	9.00 ± 0.39	10.96 ± 0.24	9.26 ± 0.35	23.29 ± 0.43	18.73 ± 0.45	23.12 ± 0.20	18.21 ± 0.22

### 3.2 Neither ELS nor PND 28–30 RU486 treatment change baseline CORT levels at PND31

**Fig. 2:**
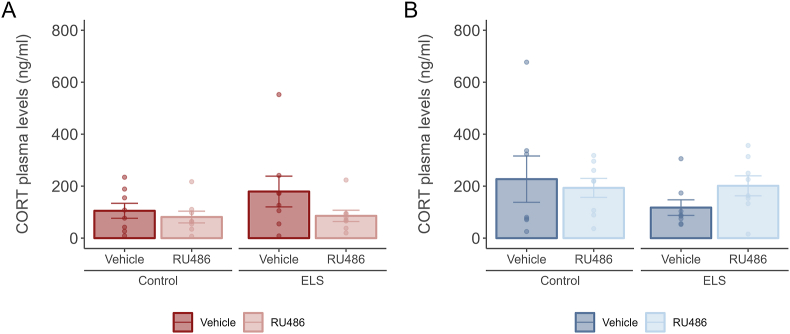
Baseline corticosterone plasma levels at PND31 were not affected by ELS or PND28-30 RU486 treatment. **A.** Barplots showing male baseline plasma corticosterone levels (in ng/ml, mean ± SEM) at PND31. No changes were found due to ELS or RU486 treatment at PND28-30. **B.** Barplots showing female baseline plasma corticosterone levels (in ng/ml, mean ± SEM) at PND31. No alterations were observed as a result of either ELS or the RU486 intervention at PND28-30. Males: N_Control-Vehicle_ = 8, N_Control-RU486_ = 8, N_ELS-Vehicle_ = 8, N_ELS- RU486_ = 8. Females: N_Control-Vehicle_ = 7, N_Control-RU486_ = 8, N_ELS-Vehicle_ = 8, N_ELS- RU486_ = 8. Males depicted in red, females in blue.

In order to validate the differential GR signalling in ELS animals and its modulation following a three-day treatment with RU486, we assessed baseline CORT levels (collected at 8:00 to measure low circadian levels), one day after the completion of the RU486 treatment (PND31). Our findings didn't reveal concurrent significant changes in circulating CORT levels one day after treatment (Fig. 2A and B).

### 3.3 ELS impairs fear memory acquisition in males but not females: prevention by RU486 treatment at adolescence

**Fig. 3:**
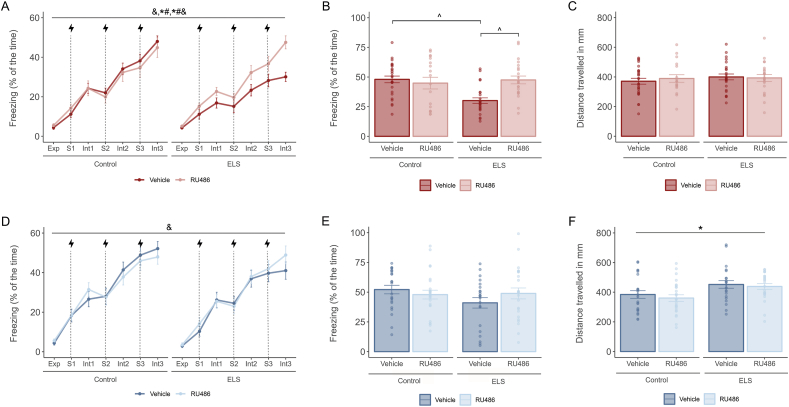
The effects of ELS and RU486 treatment in fear memory acquisition and locomotor activity in adulthood were sex dependent. **A.** Lineplots showing freezing (mean % ± SEM) across the course of conditioning in males. ELS impairs freezing acquisition over time and RU486 prevents this effect. Exp = exploration, S = shock, Int = intertrial period. **B.** Barplots showing freezing (mean % ± SEM) during the last intertrial period of the conditioning (Int3) in males. ELS animals had a reduced freezing in the last time point of the task and administration of RU486 effectively prevents this impairment. **C.** Barplots showing distance in mm (mean % ± SEM) travelled by males during the exploration time on the training day. Distance travelled was comparable between experimental groups regardless of stress and treatment condition. **D.** Lineplots showing freezing (mean % ± SEM) across the course of conditioning in females. No differences were found due to ELS or RU486 treatment. Exp = exploration, S = shock, Int = intertrial period. **E**. Barplots showing freezing (mean % ± SEM) during the last intertrial period of the conditioning (Int3) in females. No differences were found due to ELS or RU486 treatment. **F.** Barplots showing distance in mm (mean % ± SEM) travelled by females during the exploration time on the training day. Distance travelled was increased by ELS. Males: N_Control-Vehicle_ = 26, N_Control-RU486_ = 18, N_ELS-Vehicle_ = 25, N_ELS- RU486_ = 22. Females: N_Control-Vehicle_ = 23, N_Control-RU486_ = 25, N_ELS-Vehicle_ = 22, N_ELS- RU486_ = 24. Males depicted in red, females in blue. *: ELS effect. *#&: ELS by RU486 by time interaction effect. ^: post-hoc effect. Effect p ≤ 0.05.

Male mice (Fig. 3A–C) and female mice (Fig. 3D–F) were trained in an auditory fear conditioning paradigm. We first examined the percentage of freezing over time during the training (“acquisition”) phase, which we divide in exploration (Exp), paired tone-footshocks (S) and inter-trial period (Int). In male mice (Fig. 3A), we found a main effect of time (F(6.18, 550.41) = 113.375, p < 0.001) and an interaction effect between ELS and RU486 (F(1, 87) = 4.259, p = 0.042) and ELS, RU486 and time (F(6.20, 539.58) = 2.532, p = 0.019). Post-hoc analysis revealed that ELS reduced freezing during acquisition (F(1, 508) = 31.014, p < 0.001), and that RU486 prevented this overall performance effect (F(1, 468) = 25.522, p < 0.001). At the last timepoint of acquisition (Fig. 3B) we found an ELS and RU486 interaction effect (F(1, 87) = 9.867, p = 0.002). Post-hoc analysis showed that ELS at this time point significantly decreased freezing levels (F(1, 49) = 24.061, p < 0.001) and that RU486 restored ELS freezing impairment (F(1, 45) = 19.457, p < 0.001). In females (Fig. 3D and E), we observed an increase in freezing behavior over time (F(5.89,541.74) = 107.716, p < 0.001) but neither ELS nor RU486 affected freezing during acquisition.

Since general locomotor activity can affect freezing behavior, and may be affected by ELS, we also assessed the distance travelled during the exploration period before application of tone/footshock (Fig. 3C,F). Locomotor activity of male mice was not affected by either ELS or RU486 treatment (Fig. 3C). In female mice (Fig. 3F), ELS increased the distance moved (F(1,90) = 9.851, p = 0.002). Taken together, the data indicate that ELS reduced fear acquisition in male mice, which was prevented by RU486 treatment at adolescent age.

### 3.4 ELS reduces contextual freezing in male and female mice

**Fig. 4:**
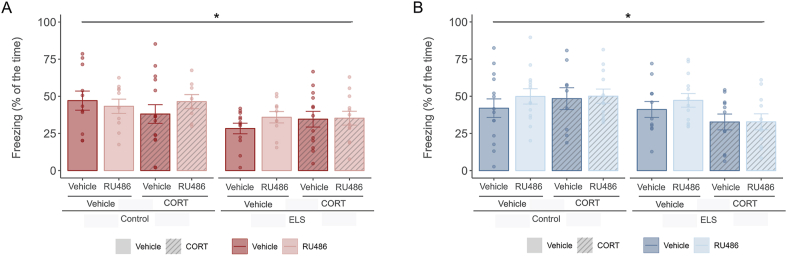
ELS decreased freezing levels in Context A at 24 h after training. **A.** Barplots showing freezing (mean % ± SEM) in males when placed back in the training context 24 h after fear conditioning. ELS males froze less than control mice. **B.** Barplots showing freezing (mean % ± SEM) in females when placed back in the training context 24 h after fear conditioning. Similarly, ELS decreased contextual freezing in females. Males: N_Control-Vehicle- Vehicle_ = 11, N_Control-RU486- Vehicle_ = 10, N_Control-Vehicle-CORT_ = 15, N_Control-RU486- CORT_ = 8, N_ELS-Vehicle- Vehicle_ = 12, N_ELS-RU486- Vehicle_ = 10, N_ELS-Vehicle-CORT_ = 13, N_ELS-RU486- CORT_ = 12. Females: N_Control-Vehicle- Vehicle_ = 14, N_Control-RU486- Vehicle_ = 13, N_Control-Vehicle-CORT_ = 9, N_Control-RU486- CORT_ = 12, N_ELS-Vehicle- Vehicle_ = 11, N_ELS-RU486- Vehicle_ = 13, N_ELS-Vehicle-CORT_ = 11, N_ELS-RU486- CORT_ = 11. Males depicted in red, females in blue.*: ELS effect. Effect p ≤ 0.05.

Twenty-four hours after training, animals were placed in the training context and mice were monitored for 3 min to assess the contextual memory, scored by freezing as percentage of time. In male mice (Fig. 4A), we found a main effect of ELS towards reduced freezing (F(1,83) = 7.191, p = 0.009). Neither corticosterone treatment after training, nor RU486 treatment during adolescence altered freezing behavior, in any of the experimental groups. In female mice (Fig. 4B), we also found a main effect of ELS on contextual freezing (F(1,86) = 5.359, p = 0.023). Neither corticosterone administration after training, nor RU486 treatment at adolescent age affected freezing in any of the experimental groups.

**Fig. 5:**
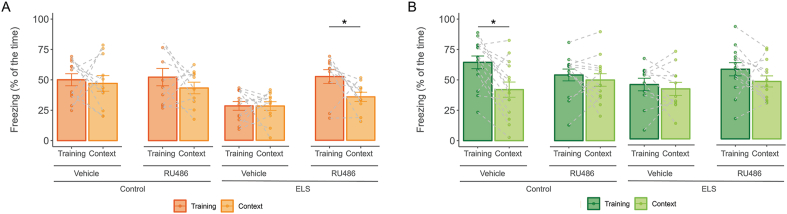
**Changes in freezing levels during acquisition and contextual retrieval explained presence/lack of effects of ELS/RU486.** **A.** Barplots showing freezing (mean % ± SEM) in males during the last timepoint of training and average performance while placing back in Context A at 24 h after training. Males maintained similar freezing levels with exception of the ELS RU486 group, that forgets 24 h later. **B.** Barplots showing freezing (mean % ± SEM) in females during the last timepoint of training and average performance while placing back in Context A at 24 h after training. Control vehicle females decreased freezing levels upon reexposure to Context A. For this comparison, to avoid any cofounding effects of post training administration of corticosterone, only the vehicle animals were paired tested. Males: N_Control-Vehicle- Vehicle_ = 11, N_Control-RU486- Vehicle_ = 10, N_ELS-Vehicle- Vehicle_ = 12, N_ELS-RU486- Vehicle_ = 10. Females: N_Control-Vehicle- Vehicle_ = 14, N_Control-RU486- Vehicle_ = 13, N_ELS-Vehicle- Vehicle_ = 11, N_ELS-RU486- Vehicle_ = 13. Males depicted in orange, females in green. *: paired significant difference. Effect p ≤ 0.05.

We next compared average freezing levels at the last timepoint of the training session with contextual retrieval 24 h later. In male mice (Fig. 5A), paired analysis revealed that freezing levels at the end of the acquisition period were comparable with freezing levels during retrieval 24 h later. Male ELS animals treated with RU486 showed less freezing during contextual retrieval 24 h later (t(9) = 2.415, p = 0.039). In female mice (Fig. 5B), control animals showed decreased freezing behavior during context retrieval (t(13) = 3.735, p = 0.003). No effects were observed in the other experimental groups.

### 3.5 ELS reduces freezing behavior during cue-exposure in male and female mice: prevention by RU486 in female mice

**Fig. 6:**
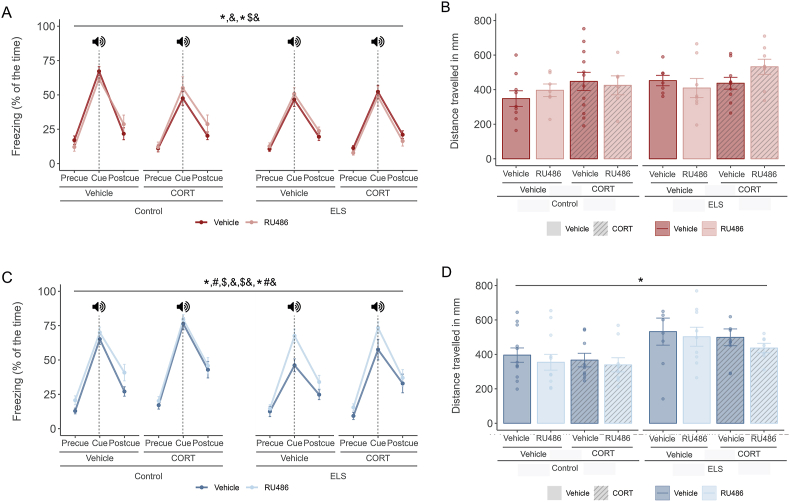
**Sex dependent effects of ELS and RU486 and CORT treatment in cue retrieval and locomotor activity in Context B at 48 h after training.** **A.** Lineplots showing freezing (mean % ± -SEM) in males across the course of cue retrieval task, divided in precue phase, cue presentation and postcue. Males froze specifically when the cue is played but ELS males freeze significantly less over the cue period. **B.** Barplots showing distance in mm (mean % ± SEM) travelled by males during the exploration time during the cue retrieval task on context B. Distance was comparable regardless of stress and treatment condition. **C.** Lineplots showing freezing (mean % ± -SEM) in females across the course of cue retrieval task, divided in precue phase, cue presentation and postcue. Females froze specifically when the cue is played but ELS males freeze significantly less over the cue period. However, ELS animals that received RU486 injection over adolescent froze comparable to control animals. In addition, CORT enhanced freezing in interaction with time. **D.** Barplots showing distance in mm (mean % ± SEM) travelled by females during the exploration time during the cue retrieval task on context B. Distance travelled was increased by ELS. Males: N_Control-Vehicle- Vehicle_ = 9, N_Control-RU486- Vehicle_ = 7, N_Control-Vehicle-CORT_ = 12, N_Control-RU486- CORT_ = 6, N_ELS-Vehicle- Vehicle_ = 7, N_ELS-RU486- Vehicle_ = 8, N_ELS-Vehicle-CORT_ = 10, N_ELS-RU486- CORT_ = 8. Females: N_Control-Vehicle- Vehicle_ = 12, N_Control-RU486- Vehicle_ = 12, N_Control-Vehicle-CORT_ = 9, N_Control-RU486- CORT_ = 9, N_ELS-Vehicle- Vehicle_ = 8, N_ELS-RU486- Vehicle_ = 9, N_ELS-Vehicle-CORT_ = 10, N_ELS-RU486- CORT_ = 7. Males depicted in red, females in blue. *: ELS effect, $: CORT effect, &: time effect, $&: CORT by time interaction effect, *$&: ELS by CORT by time interaction effect, *#&: ELS by RU486 by time interaction effect. Effect p ≤ 0.05.

At 48 h after training, mice were exposed to a novel context (Context B) for 3 min to assess freeing behavior in a novel context (pre-cue) (Fig. 6 A and C). Next, the tone was played for 30 s (cue) followed by a 1-min non-tone period (post-cue). Freezing was scored and expressed as percentage of time. In male mice (Fig. 6A), we found a main effect of time (F(1.62, 95.64) = 358.589, p < 0.001), an ELS effect (F(1,59) = 5.201, p = 0.026) and an interaction between ELS, time and CORT (F(1.62, 95.64) = 4.026, p = 0.029). Post-hoc analysis revealed a significant reduction of freezing in ELS mice during the cue phase (F(1, 72) = 10.635 p = 0.018). In females (Fig. 6C), we found a main effect of time (F(2,136) = 414.987, p < 0.001), an ELS effect (F(1,68) = 9.660, p = 0.003), a RU486 effect (F(1,68) = 9.883, p = 0.002) and a CORT effect (F(1,68) = 5.654, p = 0.020), as well as two interaction effects: CORT and time (F(2,136) = 3.671, p = 0.028), and ELS, RU486 and time (F(2,136) = 3.329, p = 0.039). Post-hoc analysis revealed less freezing in ELS mice (F(1, 72) = 9.282, p = 0.027) and increased freezing upon RU486 treatment (F(1, 72) = 9.585, p = 0.027) during the cue phase.

**Fig. 7:**
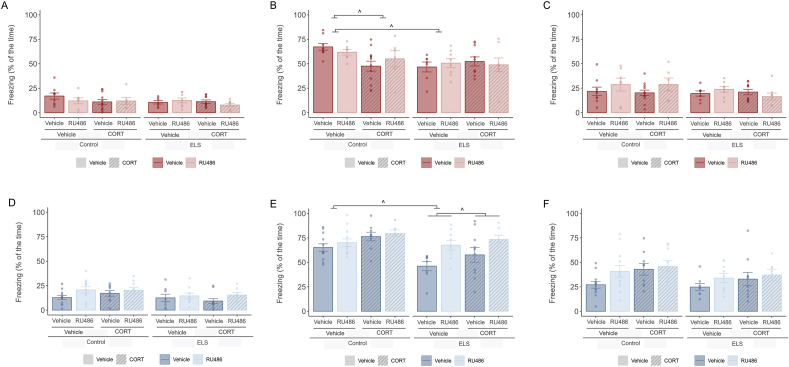
**Sex dependent effects of ELS and RU486 and CORT treatment in cue retrieval in Context B at 48 h after training – split by timepoint.** **A.** Barplots showing freezing (mean % ± -SEM) in males during the exploration of Context B – precue phase. No difference in freezing levels due to ELS, RU486 or CORT treatment. **B.** Barplots showing freezing (mean % ± -SEM) in males during the presentation of the tone – cue phase. ELS animals treated with vehicle froze significantly less. Post-training administration of CORT decreased freezing levels in non-stressed animals. **C.** Barplots showing freezing (mean % ± -SEM) in males during the recovery period – postcue phase. No difference in freezing levels due to ELS, RU486 or CORT treatment. **D.** Barplots showing freezing (mean % ± -SEM) in females during the exploration of Context B – precue phase. No difference in freezing levels due to ELS, RU486 or CORT treatment. **E.** Barplots showing freezing (mean % ± -SEM) in females during the presentation of the tone – cue phase. ELS animals treated with vehicle froze significantly less. RU486 intervention in adolescence prevented the ELS-impairment in freezing. **F.** Barplots showing freezing (mean % ± -SEM) in females during the recovery period – postcue phase. No difference in freezing levels due to ELS, RU486 or CORT treatment. Males: N_Control-Vehicle- Vehicle_ = 9, N_Control-RU486- Vehicle_ = 7, N_Control-Vehicle-CORT_ = 12, N_Control-RU486- CORT_ = 6, N_ELS-Vehicle- Vehicle_ = 7, N_ELS-RU486- Vehicle_ = 8, N_ELS-Vehicle-CORT_ = 10, N_ELS-RU486- CORT_ = 8. Females: N_Control-Vehicle- Vehicle_ = 12, N_Control-RU486- Vehicle_ = 12, N_Control-Vehicle-CORT_ = 9, N_Control-RU486- CORT_ = 9, N_ELS-Vehicle- Vehicle_ = 8, N_ELS-RU486- Vehicle_ = 9, N_ELS-Vehicle-CORT_ = 10, N_ELS-RU486- CORT_ = 7. Males depicted in red, females in blue. ^: post-hoc effect. Effect p ≤ 0.05.

Further analysis included individual phase testing (Fig. 7A–F). We found effects exclusively during the cue period for both sexes. In males (Fig. 7B), there was an ELS effect (F(1, 59) = 4.783, p = 0.033) and a stress and CORT interaction (F(1, 59) = 4.015, p = 0.050). Post-hoc analysis showed that CORT decreases freezing levels specifically in the control group (F(1, 32) = 8.584, p = 0.012). In females (Fig. 7E), we found an ELS (F(1, 68) = 11.302, p = 0.001), RU486 (F(1, 68) = 10.801, p = 0.002) and CORT (F(1, 68) = 7.539, p = 0.008) effect, as well as an interaction effect between ELS and RU486 (F(1, 68) = 4.487, p = 0.038). Post-hoc analysis showed that ELS decreased freezing levels during the cue phase (F(1, 37) = 10.141, p = 0.006) and RU486 restore this ELS deficit (F(1, 32) = 8.896, p = 0.010).

**Fig. 8:**
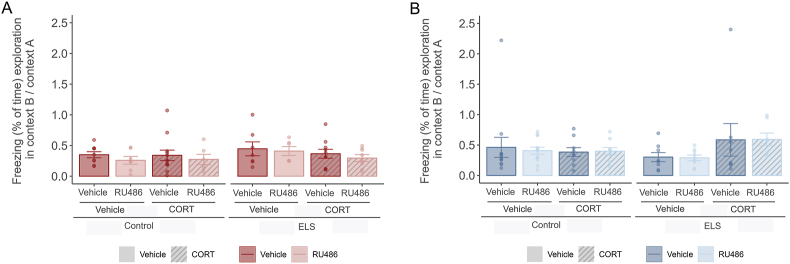
**No effects of ELS or treatment in the relative freezing of Context B in relation to Context A – proxy for generalization.** **A.** Barplots showing freezing ratio (mean % ± -SEM) in males calculated by comparing the freezing during exploration in Context B (Precue) with overall freezing in Context A during the contextual retrieval task. There were no differences due to ELS, RU486 or CORT treatment. **B.** Barplots showing freezing ratio (mean % ± -SEM) in females calculated by comparing the freezing during exploration in Context B (Precue) with overall freezing in Context A during the contextual retrieval task. There were no differences due to ELS, RU486 or CORT treatment. Males: N_Control-Vehicle- Vehicle_ = 9, N_Control-RU486- Vehicle_ = 7, N_Control-Vehicle-CORT_ = 12, N_Control-RU486- CORT_ = 6, N_ELS-Vehicle- Vehicle_ = 7, N_ELS-RU486- Vehicle_ = 8, N_ELS-Vehicle-CORT_ = 10, N_ELS-RU486- CORT_ = 8. Females: N_Control-Vehicle- Vehicle_ = 12, N_Control-RU486- Vehicle_ = 12, N_Control-Vehicle-CORT_ = 9, N_Control-RU486- CORT_ = 9, N_ELS-Vehicle- Vehicle_ = 8, N_ELS-RU486- Vehicle_ = 9, N_ELS-Vehicle-CORT_ = 10, N_ELS-RU486- CORT_ = 7. Males depicted in red, females in blue.

In addition, we analysed contextual fear generalization, which was quantified as the ratio of context-evoked freezing during exploration in context B (novel context) relative to context A (training context) (Fig. 8). We did not find changes in this ratio due to ELS, RU486 or CORT treatment, neither in males (Fig. 8A) or females (Fig. 8B). Locomotor activity during the exploration time in Context B was assessed since it may interfere with freezing levels (Fig. 6B–D). Locomotor activity of male mice was not affected by either ELS or RU486 treatment (Fig. 6B). In female mice there was an increase of distance moved in the ELS group (F(1,68) = 13.34, p < 0.001) (Fig. 6D).

These results indicate that, in males, ELS decreased freezing behavior upon auditory cue presentation, and CORT decreased freezing behavior in particular in control groups. In females, ELS decreases freezing specifically towards the tone and CORT enhances overall freezing. The specific effects of ELS during the tone presentation in females can be prevented by RU486.

### 3.6 Female freezing levels are not affected by their estrous cycle phase

Hormonal fluctuations due to the estrous cycle in females might affect their performance during the fear conditioning task (Wood et al., 2001).Therefore, we further check the freezing of the females in the fear conditioning task (the three paradigms) as well as the locomotor activity accounting for the estrous phase determined at the end of the three-day behavioral task. However, we did not find a modulatory role of the estrous cycle when we compare the models using and not using cycle as a random factor.

### 3.7 Sex differences

Finally, in order to correctly report sex differences, we conducted a combined analysis of both males and females, which yielded several significant sex-dependent findings (see also Supplementary Material and Methods for the analysis using sex as a variable). Firstly, there was a marked differential body gain from P9 until adolescence (F(1, 67) = 17.474, p < 0.001). Additionally, significant differences in body weight were observed during adolescence (F(1, 67) = 14.353, p < 0.001) and at P60 (F(1, 66) = 201.850, p < 0.001). On PND31, following a three-day RU486 treatment, the measurement of CORT revealed elevated basal levels in females (F(1, 56) = 0.903, p = 0.018). Furthermore, a trend in the data suggested a potential interaction among ELS, RU486 treatment, and sex (F(1, 56) = 3.279, p = 0.076).Regarding behavior, an interaction between sex and time during the training phase was identified (F(4.11, 727.58) = 4.324, p = 0.002), with both sexes showing learning effects, but the effect being more significant in males (F(6, 630) = 81.323, p < 0.001) compared to females (F(6, 651) = 76.428, p < 0.001). Furthermore, during auditory retrieval, an interaction of sex with time was observed, with both groups remembering, but the effect was more pronounced in females (F(2, 225) = 223.594, p < 0.001) compared to males (F(2, 198) = 215.930, p < 0.001). Notably, these sex differences only occurred during the cue (F(1, 141) = 23.799, p < 0.001) and post-cue (F(1, 141) = 28.831, p < 0.001) phases, while the exploration pre-cue was similar in both sexes. Finally, the sex analysis revealed that treatments had differential effects on males and females during the auditory retrieval. RU486 had a significant effect in females (F(1, 226) = 5.289, p = 0.044) but not in males, whereas CORT had significantly different effects in males compared to females (F(1, 211) = 19.142, p < 0.001), highlighting the sex-specific responses to these treatments.

## 4 Discussion

The present study was designed to investigate 1) whether ELS alters contextual and auditory memory in male and female mice, 2) whether such effects are moderated by post-training administration of corticosterone and 3) whether treatment with the glucocorticoid receptor antagonist RU486 during adolescence period can mitigate ELS effects later in life. Our data indicates that ELS impairs acquisition -and consequently-retrieval of contextual and auditory cue memories in male mice, whereas in females ELS primarily impacts the retrieval of both contextual and auditory cue memories. Post training administration of CORT had sex-dependent effects. In males, CORT reduced freezing behavior only during the tone presentation, an effect that was observed only in control animals. In contrast, CORT increased freezing behavior in females during the overall auditory memory retrieval task. Finally, treating mice with RU486 during their adolescent stage had preventive effects. It prevented the negative impact of ELS on memory acquisition in males and in females it prevented the ELS specific auditory memory deficits.

### 4.1 Behavioral effects of ELS in fear learning and memory: influence of sex

While the effects of ELS on fear memory have been explored previously (Arp et al., 2016; Atsak et al., 2018; Chocyk et al., 2014; Cowan et al., 2013; Fuentes et al., 2018; Lesuis et al., 2019; Richardson et al., 2016), the majority of studies have primarily focused on parameters such as retrieval, generalization, or extinction. However, fewer studies have investigated the impact of ELS on the initial acquisition phase of fearful memories. Here, we report that ELS reduces fear acquisition in male mice, but not in female mice, which is in line with previous studies on maternal separation showing deficiencies in acquisition during fear conditioning (Stevenson et al., 2009). This behavioral outcome is also in line with findings showing that ELS impairs long-term potentiation (LTP) (Brunson et al., 2005; Ivy et al., 2010; Wang et al., 2011; Lesuis et al., 2019) which is a cellular correlate of learning (Johnston, 1997; Siegelbaum and Kandel, 1991) and which could underlie the ELS-induced encoding deficits. This data might therefore suggest that difficulties in memory formation in males following ELS may, at least in part, stem from reduced synaptic plasticity and impaired memory encoding. Interestingly, in females, ELS deficits are limited to the retrieval of context and auditory cue memories and no effects were found during fear acquisition. This sex-dependent effect of ELS on acquisition has also been reported at the synaptic level where ELS enhances, rather than decreases LTP in adulthood (Derks et al., 2016).This suggests sex-specific differences in the mechanisms underlying fear acquisition. Further research in this area is necessary to gain a deeper understanding of ELS on sex-specific cellular mechanisms for fear memory learning.

Moreover, we found that ELS reduced contextual and auditory cue retrieval. This aligns with previous research conducted by our lab (Lesuis et al., 2019) and others (for a review, Alves et al., 2022). Furthermore, our study extends to these findings that the ELS effects are present in both male and female mice. In contrast, other studies using LBN as ELS model have found in females no effect of ELS (Pillai et al., 2018) or an enhancement of freezing specifically in safe context and no effect during the tone presentation (Arp et al., 2016). Differences between studies in the animal strains, LBN procedure, parameters for behavioral testing (such as intensity of the shock or number of shocks) may account for the differential findings. It is important to consider these factors when interpreting the results of ELS studies and to replicate findings across different models and experimental conditions.

Ultimately, we did not detect any alterations attributed to ELS, CORT or adolescent intervention in the context of generalization, whose characteristics can be extrapolated to PTSD symptoms. These results differ from other studies that reported enhanced fear generalization due to ELS (Elliott and Richardson, 2019; Dixon, 2023). The limitations of our experimental design impede to conduct extinction trials, which would represent another feature with clinical relevance. However, other studies have already reported impaired extinction due to ELS in maternal deprivation/maternal separation (MD/MS) models (Callaghan and Richardson, 2012; Cowan et al., 2013).

The observed reduced freezing levels in ELS males cannot be explained by ELS effects on mobility which were absent in males. However, in females we found that ELS increased mobility which may have been a confounding factor for the decreasing freezing behavior in this group. Alterations in ELS-induced nociception that might affect the perception of the footshock are unlikely since LBN has been reported to increase somatic sensitivity (Green et al., 2011; Prusator et al., 2016) indicating that ELS animals do sense the foot-shock (even more than controls). These observations reinforce the notion that the behavioral changes observed are predominantly rooted in cognitive impairments rather than physical responses to the footshock stimulus.

There are multiple interpretations of the ELS effects on fear memory. The simplest one, is that our LBN model modifies cognitive function, in concordance with multiple studies in spatial and emotional learning (for an extensive review see Walker et al., 2017). In contrast, one might argue that the fear conditioning paradigm served as a second emotional stressor. In that view, ELS-induced reduction of freezing levels, which may allow mice for a more active behavioral response that can have adaptive value (Santarelli et al., 2017). More ecologically designed studies will require whether and how ELS alters coping mechanisms to serve adaptive responses.

### 4.2 CORT effects: modulation of ELS effects on auditory memory

Corticosterone has significant effects on fear memory, including the enhancement of memory consolidation (Abrari et al., 2009; Den et al., 2014; Hui, 2004; Maheu et al., 2004; Roozendaal and McGaugh, 1997; Roozendaal et al., 2006; Sandi and Rose, 1997a, 1997b; van Ast et al., 2013; Xiong et al., 2015), extinction (de Quervain et al., 2019; Den et al., 2014; Gourley et al., 2009) and generalization (Bahtiyar et al., 2020; dos Santos Corrêa et al., 2021; Gazarini et al., 2022; Lesuis et al., 2021). Here, our aim was to investigate whether the effects of post-training administration of CORT on conditioning could be modulated by ELS and RU486.

In our studies we did not find that CORT altered contextual memory. One possible explanation is that animals showed already substantial freezing at 24 h after training, potentially inducing a ceiling effect that could mask CORT effects. Notably, CORT has been reported to heighten contextual freezing in particular when compared to control conditions with lower freezing levels (Xiong et al., 2015). Likewise, CORT did not alter contextual generalization, an effect that was also absent in ELS animals. While CORT has been reported to increase memory generalization (Bahtiyar et al., 2020; dos Santos Corrêa et al., 2021; Gazarini et al., 2022; Lesuis et al., 2021) possible effects may have been obscured due to differences in experimental design, paradigm and treatment doses. Careful designed studies will be required to test whether ELS alters memory generalization and whether ELS alters effects and sensitivity of CORT to evoke memory generalization.

We did find a sex-opposite effect of CORT during the auditory cue retrieval. We found an overall increase in freezing behavior induced by CORT in females, which aligns with previous research indicating that females tend to be more responsive to CORT (Logrip et al., 2017).

In contrast, CORT decreased freezing only in the control group in males when analyzing the cue presentation. This could be explained since the ELS animals have already lower freezing levels than cannot be further modulated by CORT. However, the differential effects of CORT treatment in males with or without ELS raise intriguing questions about underlying mechanisms. Functionally, ELS males exhibit altered sensitivity to CORT compared to control males. For instance, the hippocampal NMDA/AMPA ratio is diminished in ELS males, and this alteration can be rectified by corticosterone, without affecting the NMDA/AMPA ratio in control animals (Pillai et al., 2018). Importantly, these effects are region-specific, as they were observed in the hippocampus but not in the amygdala (Pillai et al., 2018). Similarly, in a maternal separation model of ELS, acute CORT administration enhances LTP in the medial prefrontal cortex of adolescent control rats, but this effect is not observed in rats with a history of MS (Majcher-Maślanka et al., 2018). Whether and how these differential sensitivity to CORT upon ELS relate to our behavioral effects presents an interesting avenue for investigation.

Taken together, we conclude that CORT has sex-dependent effects on freezing behavior during auditory cue retrieval, and these effects are not modulated by a history of ELS or RU486 intervention. Future studies may be needed to further address thresholds at which CORT may alter other types of memory and/or memory generalization and its modulation by ELS or RU486.

### 4.3 RU486 in adolescence: partial prevention of ELS effects

Earlier studies have reported that treatment with RU486 at adolescence mitigates later life effects of ELS (Arp et al., 2016; Loi et al., 2017b). We therefore further characterized whether blocking GRs at adolescent age was able to mitigate later life effects of ELS on contextual and auditory cue retrieval in male and female mice.

The adolescent period was chosen since it constitutes a period of high brain plasticity (Fuhrmann et al., 2015) and may have translational relevance for treating individuals once ELS has taken place. In particular, we choose to intervene from PND 28–30 since it is known that this period is a developmental window important for the stress axis development (Romeo et al., 2016). Also, there is evidence that maturation of fear circuits is accelerated by the LBN procedure during this time frame (Bath et al., 2017). We used RU486, a glucocorticoid antagonist that is being shown to be effective to prevent the behavioral effects after chronic stressors (Ding et al., 2020; Papilloud et al., 2019) and also after ELS (Arp et al., 2016; Loi et al., 2017b). We used a 3-day injection protocol since there is evidence that already one day (Hu et al., 2012; Mayer et al., 2006) and four days (Mayer et al., 2006) of injections are effective to reverse chronic stress-induced effects. This was encouraged by the fact that 3 days RU486 treatment during this period was proven to be effective to prevent spatial memory effects in other models of ELS such as maternal deprivation (Loi et al., 2017b).

We found that RU486 intervention prevented ELS effects in a sex-dependent manner. In males, the intervention was effective during the acquisition phase, whereas in females, RU486 prevented ELS effects when only examining the tone presentation. Contextual freezing was not affected by RU486 intervention.

The partial effects of RU486 on ELS memory impairments suggest that that alterations in GR signaling partially drive ELS memory effects. LBN has been reported to modify the HPA axis. However, the transient and sometimes challenging-to-replicate nature of these effects raise uncertainties (e.g., elevated CORT levels at PND9 after ELS reported in Naninck et al., 2015; Rice et al., 2008 but not observed in Bath et al., 2016; Brosens et al., 2023b; Sachs et al., 2013). Similarly, while others found enhanced corticosterone levels in ELS exposed male mice at adolescent age (Hsiao et al., 2016) we did not observe this effect. Previous work in the lab (Arp et al., 2016) found no differences in hippocampal GR protein levels between ELS and control conditions at PND28 in males. These levels remained unaltered by ELS or RU486 treatment in adulthood. However, an increase in adrenal size at PND28 upon ELS suggests modified downstream HPA axis regulation, potentially explaining the partial efficacy of our antagonist intervention.

RU486, administered during adolescence, has been shown to normalize the behavioral effects of ELS in previous studies (Arp et al., 2016; Loi et al., 2017b) and also partially in our current study. However, the underlying mechanisms remain unexplored. These mechanisms may involve modulation of stress-induced corticosteroid levels (which we currently overlook in baseline assessments), and actions through other components of the HPA axis, such as adaptive changes of the mineralocorticoid receptor or the potential involvement of other modulators like CRH (Carroll et al., 1996). The effects of RU486 may also involve effects on targets that not exclusively altered by ELS. Alternative hypotheses include but not limit to changes at other levels (e.g. RNA), in other brain regions or via other mechanisms (such as chromatin accessibility, unpublished data).

The sex-dependent effect of RU486 on auditory retrieval may be related to dual action of RU486, which is also a progesterone receptor antagonist (McDonnell and Goldman, 1994). Progesterone has been shown to enhance or dampen fear memories during acquisition, extinction, and testing (Barros et al., 2015; Frye and Walf, 2004; Hiroi and Neumaier, 2006) with the amygdala being particularly responsive to its influence (van Wingen et al., 2008). This could partially explain female-specific effects of RU486 during auditory retrieval, which is a more amygdala-dependent task (Phillips and LeDoux, 1992). Finally, transient blockage of progesterone and glucocorticoid receptors between PND 28–30, a period that overlaps with puberty and sexual maturation (Schneider, 2013) may explain potentially the sex-dependent effects. Exploring more specific GR-antagonists (Buurstede et al., 2022; Viho et al., 2019) in the future could provide valuable insights into the impact of GCs signaling on memory processing and its potential modulatory role upon ELS.

### 4.4 Estrous cycle doesn't modify freezing levels in females

Various lines of evidence suggest the estrous cycle can modify the performance of females in the fear conditioning paradigm (Carvalho et al., 2021). Therefore, we did run a comparison of linear models with and without including estrous cycle as a variable. These results showed no differences in our model, meaning that females performed the same independently of their estrous status. It is important to realize that we made a distinction between estrus and non-estrus phase and, with approximately 10 animals per group, there may have been a lack of statistical power to make conclusive claims. For that, a larger sample size would be required, which we did not implement since this question was beyond the scope of our study.

## 5 Conclusion

We report that ELS (LBN from PND 2–9) leads to long-lasting and sex-dependent changes on learning and memory. In males, ELS impairs the ability to acquire fear, which may affect the retrieval of both contextual and cue-associated memories. In contrast, in females, ELS only impairs the retrieval of contextual and auditory cue memories. Post-training administration of CORT enhanced freezing responses in females across the entire auditory retrieval process whereas in males only in the control group and solely during tone presentation. Intervention with RU486 during the adolescence mitigates the impact of ELS on acquisition in males. In females, RU486 exclusively prevented ELS-induced effects on auditory cue retrieval. Further research will be required to elucidate the neural mechanisms underlying this ELS impairment, which is crucial for understanding the sensitivity to develop cognitive impairments later in life, as well as the long-lasting protective factors that RU486 intervention can have in such disorders.

## Funding

This research was supported by a personal grant from Fundación Mutua Madrileña to 10.13039/100013118JSG (BP173352019) and via the Swammerdam Institute for Life Sciences (SILS). HJK. is supported by the Memorabel Dementia Program of ZonMW (Mechanisms of Dementia [MODEM]), 10.13039/501100010969Alzheimer Nederland (WE.03-2017-01; WE.03-2023-15), 10.13039/501100019541Amsterdam Brain and Cognition (10.13039/501100019541ABC, 15PG20), and the Center for Urban Mental Health, a Research Priority Area at the 10.13039/501100001827University of Amsterdam.

## CRediT authorship contribution statement

**Jeniffer Sanguino-Gómez:** Conceptualization, Data curation, Funding acquisition, Formal analysis, Investigation, Methodology, Validation, Visualization, Writing – original draft, Writing – review & editing. **Harm J. Krugers:** Funding acquisition, Project administration, Resources, Supervision, Writing – original draft, Writing – review & editing.

## Declaration of competing Interest

The authors declare no conflicts of interest.

## Data Availability

Data will be made available on request.
